# Crystal structure, thermal and fluorescence properties of 2,2′:6′,2′′-terpyridine-1,1′,1′′-triium tetra­chlorido­nickelate(II) chloride

**DOI:** 10.1107/S2056989017016784

**Published:** 2017-11-28

**Authors:** Ouahida Zeghouan, Lamia Bendjeddou, Hocine Merazig, Jean Claude Daran

**Affiliations:** a Unité de Recherche Chimie de l’Environnement et Moléculaire, Structurale, ’CHEMS’, Faculté des Sciences Exactes,Campus Chaabet Ersas, Université, Frères Mentouri Constantine 1, 25000 Constantine, Algeria; b Centre de Recherche en Biotechnologie, Constantine, Algeria; c Laboratoire de Chimie de Coordination, UPR-CNRS 8241, 205 route de Narbonne, 31077 Toulouse Cedex 4, France

**Keywords:** crystal structure, nickel(II) complex, terpyridinium cation, protonation, hydrogen bonds, fluorescence

## Abstract

The title compound, (C_15_H_14_N_3_)[NiCl_4_]Cl, comprises an Ni^II^ cation tetra­hedrally coordinated by four chloride anions, a non-coordinating chloride anion and an essentially planar terpyridinium trication (tpyH_3_^3+^), in which the central pyridinium ring forms dihedral angles of 5.7 (2) and 6.0 (2)° with the peripheral pyridinium rings. Three inter-species N—H⋯Cl hydrogen bonds are formed with the Cl^−^ anion, which also forms a link between the (tpyH_3_^3+^) cations through an aromatic C—H⋯Cl inter­action, forming a zigzag chain extending along the 2_1_ (*b*) screw axis. Two of the anionic Cl atoms of the [NiCl_4_]^2−^ anions form Ni—Cl⋯π inter­actions with separate pyridinium rings [Ni⋯*Cg* = 3.669 (3) and 3.916 (4) Å]. In the crystal, successive undulating inorganic and organic layers are formed, extending across the (100) plane. Thermogravimetric and differential thermal analysis (TGA/DTA) indicate that the compound starts to decompose at 313 K and may be a candidate for use as a blue-light luminescent material.

## Abstract 2

The synthesis, and structural determination of 2,2′:6′,2′′-terpyridine-1,1′,1′′-triium tetra­chlorido­nickelate(II) chloride are reported. The crystal structure features N—H⋯Cl and C—H⋯Cl hydrogen bonds and Ni—Cl⋯π ring inter­actions.

## Chemical context   

The 2,2′:6′,2′′-terpyridine mol­ecule (tpy) has been the object of numerous studies because of its excellent complexing properties on metal ions. The multitude of applications of this cation motivated a large development in the synthesis of terpyridines during the last decade. The compounds derived from the terpyridine mol­ecule can be used in photochemistry for the realization of luminescent materials (Adeloye *et al.*, 2012 ▸), the assembly of electrochemical sensors (Indelli *et al.*, 1998 ▸), in photocatalysis (Mori *et al.*, 2012 ▸) and as a sensitizing agent in photovoltaic conversion processes (Kohle *et al.*, 1996 ▸). The literature reports some hybrid complexes of transition metal species incorporating tpy as a neutral ligand as well as complexes with its protonated forms [(tpyH^+^), (tpyH_2_^2+^), (tpyH_3_^3+^)] (Kochel, 2006 ▸). The title compound, which is a new hybrid complex, was characterized using IR spectroscopy and X-ray crystallography and its thermal and fluorescence properties have also been recorded.

## Structural commentary   

**Figure 1:**
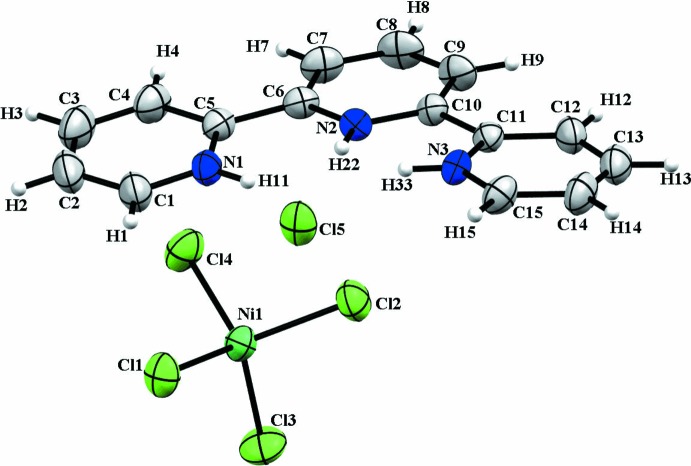
The asymmetric unit of (C_15_H_14_N_3_)[NiCl_4_]Cl, showing the atom-numbering scheme. Displacement ellipsoids are drawn at the 50% probability level.

Crystals of (C_15_H_14_N_3_)[NiCl_4_]Cl, (I), are monoclinic (space group *P*2_1_), the asymmetric unit comprising an organic terpyridinium (tpyH_3_^3+^) cation, a tetra­chloro­nickelate(II) [NiCl_4_]^2−^ dianion and a free chloride anion (Cl5) (Fig. 1 ▸).
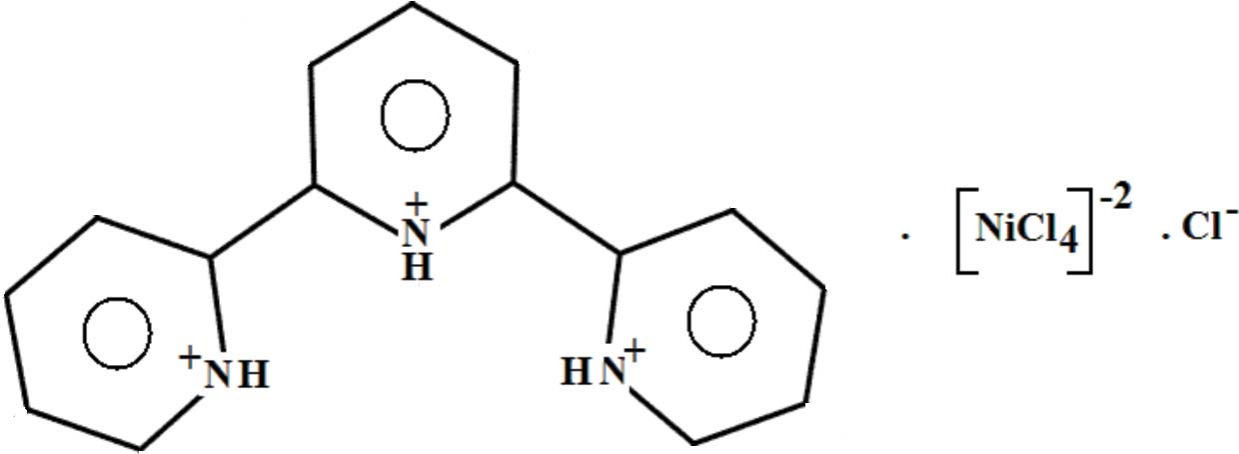


The (tpyH_3_^3+^) cation has the *cis*–*cis* conformation and is essentially planar, with dihedral angles between the central pyridine ring and the two peripheral ring moieties of the ligand of 5.7 (2) and 6.0 (2)°. The three protonated N atoms (N1, N2 and N3) form hydrogen bonds with the chloride counter-anion (Cl5) (Table 1 ▸), giving short H11⋯H22 and H22⋯H33 contacts (1.70 and 1.68 Å, respectively), which are comparable to those reported for tpyH_3_Cl(PF_6_)_2_ (H⋯H range: 1.667–1.684 Å; Yoshikawa *et al.*, 2016 ▸). The complete protonation of an aromatic mol­ecule that is nitro­gen-enriched (a polynitro­genous derivative) is rarely observed, probably because of an unfavorable charge distribution resulting from the proximity of the nitro­gen H atoms, as previously indicated in this structure. This results in an opening of the inter­nal angles of the three N atoms [C1—N1—C5 = 124.0 (4), C10—N2—C6 = 118.9 (3) and C15—N3—C11 = 123.2 (3)°]. These values are comparable to those found in the literature for (tpyH_3_^3+^). In 2,2′:6′,2′′-terpyridine­triium bis­(hexa­fluorido­phosphate) chloride (Yoshikawa *et al.*, 2016 ▸), C1—N1—C5 = 122.90, C6—N2—C10 = 117.60 and C11—N3—C15 = 123.27, C16—N4—C20 = 123.69, C21—N5—C25 = 118.22 and C26—N6—C30 = 123.97° and in *catena*-[(2,2′:6′,2′′-terpyridin­ium)(μ_3_-sulfato)­sulfato­dioxouranium) nitrate dihydrate] (Jie Ling *et al.*, 2010 ▸), C1—N1—C5 = 123.33, C6—N2—C10 = 118.03 and C11—N3—C15 = 123.29°. The inter­nal angles for a deprotonated terpyridine are C1—N1—C5 = 116.9 (8), C10—N2—C6 = 119.6 (11) and C15—N3—C11 = 117.1 (8)° (Maynard *et al.*, 2009 ▸).

**Figure 2:**
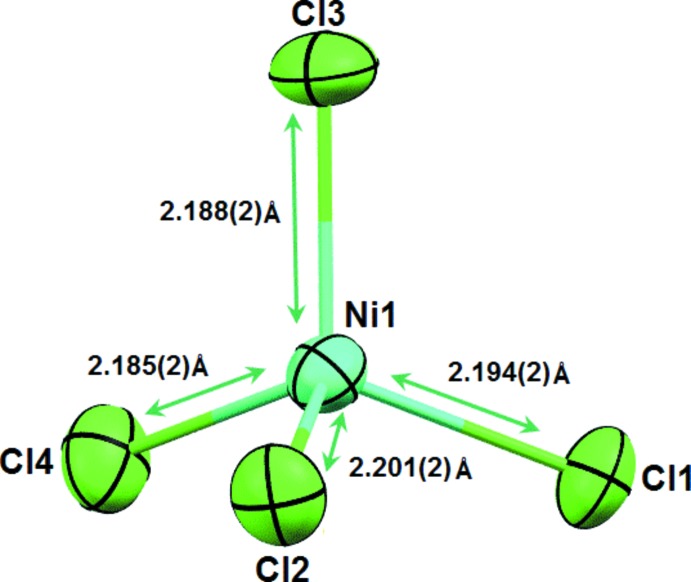
The nickel tetra­hedral environment.

The nickel(II) centre of the dianion has a quasi-regular tetra­hedral environment [Ni—Cl bond length range, 2.185 (2)–2.201 (2) Å and Cl—Ni—Cl bond angle range, 108.08 (5)–111.59 (5)°] (Fig. 2 ▸). The inter­atomic distance and angle values are in good agreement with those taken from the literature (Igashira-Kamiyama *et al.*, 2013 ▸).

## Supra­molecular features   

**Figure 3:**
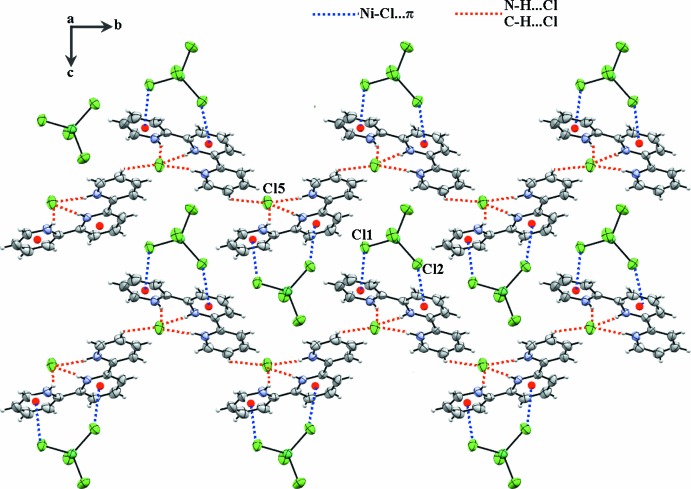
A view of the two-dimensional network of (I), showing the N—H⋯Cl and C—H⋯Cl hydrogen bonds (red dashed lines) and Ni—Cl⋯π inter­actions (blue dashed lines).

The previously described inter-species unit formed through the three individual N—H⋯Cl hydrogen bonds between the (tpyH_3_^3+^) cation and the Cl5^−^ anion (Table 1 ▸) is extended through a C14—H14⋯Cl5^i^ hydrogen bond into chains extending along the 2_1_ screw axis of the unit cell. Convoluted layers comprising successive [tpyH_3_^3+^, Cl^−^] (type *A*) and [NiCl_4_]^2−^ (type *B*) ions extend across the (100) plane (Figs. 3 ▸ and 4 ▸). Two of the anionic Cl atoms of the [NiCl_4_]^2−^ anion form Ni—Cl⋯π inter­actions with separate pyridine ring moieties of the cation within the asymmetric unit: Ni1—Cl1⋯*Cg*1 = 3.916 (4) Å and Ni1—Cl2⋯*Cg*2 = 3.669 (3) Å, where *Cg*1 and *Cg*2 are the centroids of the N1/C1–C5 and N2/C6–C10 rings, respectively (Fig. 3 ▸).

## Thermogravimetric analysis (TGA)   

Thermal analyses were performed on a SETARM 92-16.18 PC/PG 1 instrument from 303 to 1273 K under a dynamic air atmosphere and under nitro­gen at 200.0 ml min^−1^ with a heating rate of 10 K min^−1^.

**Figure 5:**
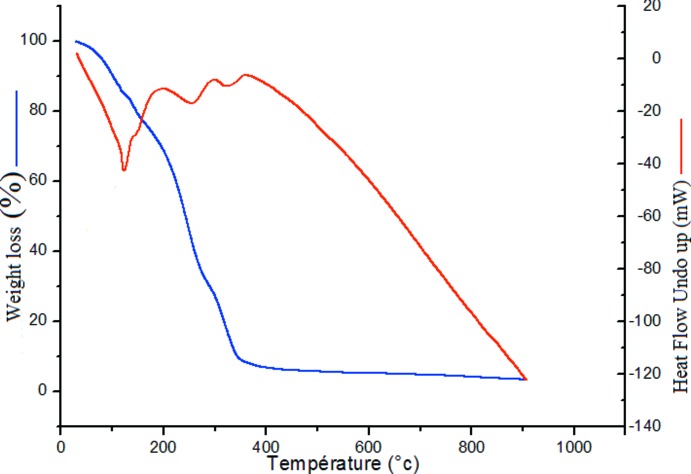
The thermogravimetric (TG) and differential thermal analysis (DTA) curves.

The stability of the (C_15_H_14_N_3_)[NiCl_4_]Cl complex was measured by TGA and the experimental results are in agreement with the calculated data. As shown in Fig. 5 ▸, the first weight loss of 16.5% (calculated 15.21%) at 40–126 K corresponds to the loss of the two coordinated chloride anions and the second loss of 48.6% (calculated 49.9%) at 126–281 K corresponds to the loss of the organic mol­ecule tpyH_3_^3+^, and then the two coordinated and free chloride anions gradually decompose (Δ*P*/*P* = 23.14%, calculated = 22.51%). In addition, the corresponding endothermic peaks (at 394.16; 554.63°C and at 638 K) in the differential scanning ATD curve also record the processes of weight loss.

## Luminescent properties   

Photoluminescence spectra were measured using a Cary Eclipse (Agilent Technologies) fluorescence spectrophotometer.

**Figure 6:**
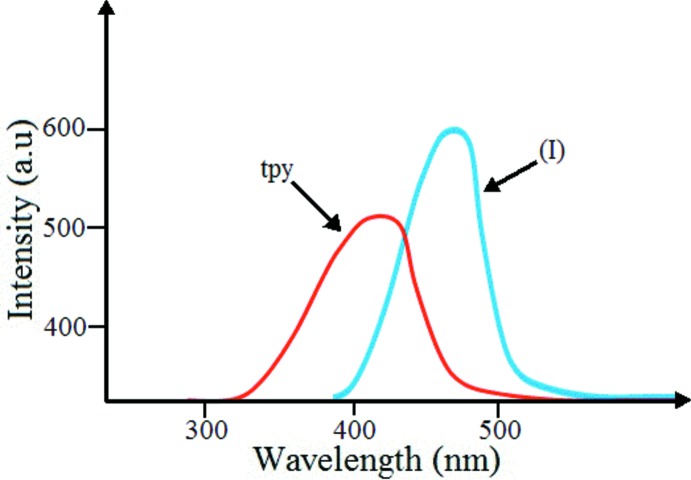
The solid-state fluorescence spectrum of tpy and the title compound (I) (excitation at 250 nm).

The fluorescence properties of (C_15_H_14_N_3_)[NiCl_4_]Cl and the free ligand tpy were investigated in the solid state at 298 K. As depicted in Fig. 6 ▸, the new compound (I) exhibits fluorescence emission at *ca* 481 nm (excited at 250 nm) compared to that of tpy (425 nm, excited at 250 nm), which can be attributed to π–π* electronic transitions. Thus, the title compound may be a candidate for use as a blue-light luminescent material and it is believed that more transition metal heterocyclic compounds with good luminescent properties may be developed (Wen *et al.*, 2007 ▸; Zhang *et al.*, 2010 ▸; Huang *et al.*, 2013 ▸).

## Database survey   

A search of the Cambridge Structural Database (Version 5.38; Groom *et al.*, 2016 ▸) shows 4279 hits comprising the terpyridine species. However, only two structures containing the (tpyH_3_^3+^) form are present (Ling *et al.*, 2010 ▸; Yoshikawa *et al.*, 2016 ▸).

## Synthesis and crystallization   

All the chemicals and solvents were purchased commercially and used as received. The infrared spectra were recorded on a Perkin–Elmer spectrometer at room temperature in the range of 4000–500 cm^−1^. tpy (1.67 g, 10 mmol) was dissolved in a 50/50 mixture of water and ethanol (20 ml) in a 50 ml round-bottom flask. Nickel(II) chloride (2.50 g, 10 mmol) was added to the flask to give a green-coloured solution that was stirred for 3 h under gentle heat, producing a green-coloured precipitate. The precipitate was filtered and washed twice with cold water/ethanol solvent then dried under vacuum for 20 min, producing a green powder (2.7g, 64% yield). Green prismatic crystals of the title complex (I) suitable for X-ray analysis were obtained from water/ethanol solvent. IR of (I) (cm^−1^): 3390 (*v*/*s*), 2930 (*v*/*s*), 1667.8 (*s*), 1622.4 (*s*), 1417.4 (*m*), 987.6 (*w*), 540.6 (*w*).

## Refinement   

Crystal data, data collection and structure refinement details are summarized in Table 2 ▸. All H atoms were placed at calculated positions and refined as riding atoms, with C—H = 0.93 Å, N—H = 0.86 Å and with *U*_iso_(H) = 1.2*U*_eq_(C,N). Although not of relevance with this achiral mol­ecule, the Flack parameter (Flack, 1983 ▸) was determined as 0.178 (16) for 4425 Friedel pairs. Minor non-merohedral twinning was identified and allowed for in the refinement, giving a BASF factor of 0.1783.

## Supplementary Material

Crystal structure: contains datablock(s) I. DOI: 10.1107/S2056989017016784/zs2392sup1.cif

Structure factors: contains datablock(s) I. DOI: 10.1107/S2056989017016784/zs2392Isup2.hkl

CCDC reference: 1587116

Additional supporting information:  crystallographic information; 3D view; checkCIF report

## Figures and Tables

**Figure 4:**
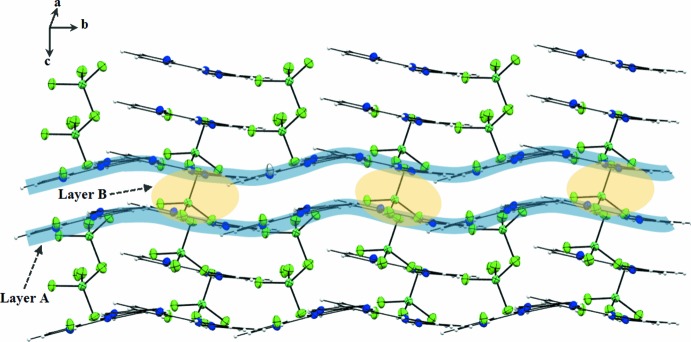
A perspective view of layers *A* and *B*.

**Table 1:** Hydrogen-bond geometry (Å, °)

*D*—H⋯*A*	*D*—H	H⋯*A*	*D*⋯*A*	*D*—H⋯*A*
N1—H11⋯Cl5	0.86	2.26	3.026 (4)	149
N2—H22⋯Cl5	0.86	2.67	3.532 (4)	178
N3—H33⋯Cl5	0.86	2.25	3.010 (4)	148
C14—H14⋯Cl5^i^	0.93	2.78	3.421 (6)	127

Symmetry code: (i) 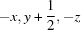.

**Table 2:** Experimental details

Crystal data	
Chemical formula	(C_15_H_14_N_3_)[NiCl_4_]Cl
*M*_r_	472.25
Crystal system, space group	Monoclinic, *P*2_1_
Temperature (K)	293
*a*, *b*, *c* (Å)	6.689 (5), 13.809 (5), 10.620 (5)
β (°)	101.271 (5)
*V* (Å^3^)	962.0 (9)
*Z*	2
Radiation type	Mo *K*α
μ (mm^−1^)	1.71
Crystal size (mm)	0.20 × 0.10 × 0.08

Data collection	
Diffractometer	Bruker APEXII CCD
No. of measured, independent and observed [*I* > 2σ(*I*)] reflections	36239, 8772, 6308
*R*_int_	0.031
(sin θ/λ)_max_ (Å^−1^)	0.828

Refinement	
*R*[*F* ^2^ > 2σ(*F* ^2^)], *wR*(*F* ^2^), *S*	0.059, 0.150, 1.15
No. of reflections	8772
No. of parameters	218
No. of restraints	1
H-atom treatment	H-atom parameters constrained
Δρ_max_, Δρ_min_ (e Å^−3^)	0.54, −0.51

Computer programs: *APEX2* and *SAINT* (Bruker, 2006 ▸), *SHELXS97* and *SHELXL97* (Sheldrick, 2008 ▸), *ORTEP-3 for Windows* (Farrugia, 2012 ▸), *Mercury* (Macrae *et al.*, 2008 ▸) and *POVRay* (Persistence of Vision, 2004 ▸).

## supplementary crystallographic information

### Crystal data

**Table:**

(C_15_H_14_N_3_)[NiCl_4_]Cl	*F*(000) = 476
*M**_r_* = 472.25	*D*_x_ = 1.630 Mg m^−^^3^
Monoclinic, *P*2_1_	Mo *K*α radiation, λ = 0.71073 Å
Hall symbol: P 2yb	Cell parameters from 6308 reflections
*a* = 6.689 (5) Å	θ = 3.0–36.1°
*b* = 13.809 (5) Å	µ = 1.71 mm^−^^1^
*c* = 10.620 (5) Å	*T* = 293 K
β = 101.271 (5)°	Prism, green
*V* = 962.0 (9) Å^3^	0.20 × 0.10 × 0.08 mm
*Z* = 2	

### Data collection

**Table:**

Bruker APEXII CCD diffractometer	6308 reflections with *I* > 2σ(*I*)
Radiation source: fine-focus sealed tube	*R*_int_ = 0.031
Graphite monochromator	θ_max_ = 36.1°, θ_min_ = 3.0°
φ and ω scans	*h* = −11→10
36239 measured reflections	*k* = −22→22
8772 independent reflections	*l* = −17→17

### Refinement

**Table:**

Refinement on *F*^2^	Primary atom site location: structure-invariant direct methods
Least-squares matrix: full	Secondary atom site location: difference Fourier map
*R*[*F*^2^ > 2σ(*F*^2^)] = 0.059	Hydrogen site location: inferred from neighbouring sites
*wR*(*F*^2^) = 0.150	H-atom parameters constrained
*S* = 1.15	*w* = 1/[σ^2^(*F*_o_^2^) + (0.0529*P*)^2^ + 0.6276*P*] where *P* = (*F*_o_^2^ + 2*F*_c_^2^)/3
8772 reflections	(Δ/σ)_max_ < 0.001
218 parameters	Δρ_max_ = 0.54 e Å^−^^3^
1 restraint	Δρ_min_ = −0.51 e Å^−^^3^

### Special details

**Table:**

Geometry. Bond distances, angles etc. have been calculated using the rounded fractional coordinates. All su's are estimated from the variances of the (full) variance-covariance matrix. The cell esds are taken into account in the estimation of distances, angles and torsion angles
Refinement. Refinement of F^2^ against ALL reflections. The weighted R-factor wR and goodness of fit S are based on F^2^, conventional R-factors R are based on F, with F set to zero for negative F^2^. The threshold expression of F^2^>2sigma(F^2^) is used only for calculating R-factors(gt) etc. and is not relevant to the choice of reflections for refinement. R-factors based on F^2^ are statistically about twice as large as those based on F, and R- factors based on ALL data will be even larger.

### Fractional atomic coordinates and isotropic or equivalent isotropic displacement parameters (Å^2^)

**Table:**

	*x*	*y*	*z*	*U*_iso_*/*U*_eq_	
N1	0.6965 (5)	0.0120 (2)	0.2808 (3)	0.0423 (8)	
N2	0.6834 (4)	0.1969 (2)	0.2073 (2)	0.0345 (7)	
N3	0.3276 (4)	0.2445 (2)	0.0620 (3)	0.0378 (8)	
C1	0.6807 (7)	−0.0822 (3)	0.3068 (4)	0.0564 (14)	
C2	0.8509 (9)	−0.1305 (4)	0.3730 (5)	0.0682 (16)	
C3	1.0290 (9)	−0.0823 (4)	0.4081 (5)	0.0704 (16)	
C4	1.0401 (7)	0.0151 (4)	0.3788 (4)	0.0570 (14)	
C5	0.8694 (5)	0.0637 (3)	0.3144 (3)	0.0400 (9)	
C6	0.8612 (5)	0.1667 (3)	0.2779 (3)	0.0377 (8)	
C7	1.0221 (6)	0.2299 (4)	0.3166 (4)	0.0514 (13)	
C8	0.9989 (6)	0.3259 (3)	0.2837 (5)	0.0566 (11)	
C9	0.8147 (6)	0.3578 (3)	0.2109 (4)	0.0510 (11)	
C10	0.6616 (5)	0.2905 (2)	0.1750 (3)	0.0360 (8)	
C11	0.4595 (5)	0.3173 (2)	0.0989 (3)	0.0374 (8)	
C12	0.3955 (7)	0.4115 (3)	0.0654 (4)	0.0493 (11)	
C13	0.1989 (7)	0.4256 (3)	−0.0041 (4)	0.0555 (14)	
C14	0.0724 (7)	0.3501 (4)	−0.0406 (4)	0.0574 (14)	
C15	0.1389 (6)	0.2582 (3)	−0.0065 (4)	0.0496 (11)	
Ni1	0.67429 (7)	0.12776 (3)	0.66208 (4)	0.0431 (1)	
Cl1	0.53022 (16)	−0.01301 (7)	0.60866 (11)	0.0556 (3)	
Cl2	0.55986 (17)	0.23114 (8)	0.50704 (10)	0.0576 (3)	
Cl3	0.6050 (2)	0.18121 (10)	0.84272 (11)	0.0672 (4)	
Cl4	1.00476 (14)	0.11245 (9)	0.68627 (12)	0.0629 (4)	
Cl5	0.27653 (15)	0.03265 (7)	0.11437 (12)	0.0576 (3)	
H1	0.55780	−0.11470	0.28090	0.0680*	
H2	0.84260	−0.19570	0.39320	0.0820*	
H3	1.14390	−0.11440	0.45200	0.0840*	
H4	1.16290	0.04800	0.40240	0.0680*	
H7	1.14480	0.20740	0.36450	0.0620*	
H8	1.10530	0.36920	0.30980	0.0680*	
H9	0.79550	0.42250	0.18730	0.0610*	
H11	0.58970	0.04130	0.24020	0.0510*	
H12	0.48240	0.46380	0.08890	0.0590*	
H13	0.15350	0.48820	−0.02590	0.0670*	
H14	−0.05810	0.36030	−0.08820	0.0690*	
H15	0.05350	0.20540	−0.03080	0.0600*	
H22	0.58510	0.15670	0.18310	0.0410*	
H33	0.36610	0.18630	0.08330	0.0450*	

### Atomic displacement parameters (Å^2^)

**Table:**

	*U*^11^	*U*^22^	*U*^33^	*U*^12^	*U*^13^	*U*^23^
N1	0.0412 (14)	0.0405 (14)	0.0434 (14)	0.0049 (11)	0.0040 (11)	0.0059 (11)
N2	0.0329 (11)	0.0359 (12)	0.0329 (11)	−0.0041 (9)	0.0023 (9)	−0.0026 (10)
N3	0.0373 (13)	0.0349 (13)	0.0394 (13)	0.0067 (10)	0.0029 (10)	−0.0032 (10)
C1	0.069 (3)	0.044 (2)	0.057 (2)	0.0067 (19)	0.014 (2)	0.0092 (17)
C2	0.099 (4)	0.050 (2)	0.059 (2)	0.021 (3)	0.024 (3)	0.017 (2)
C3	0.067 (3)	0.081 (3)	0.061 (2)	0.032 (3)	0.007 (2)	0.016 (2)
C4	0.047 (2)	0.073 (3)	0.048 (2)	0.0178 (19)	0.0019 (16)	0.0088 (19)
C5	0.0381 (15)	0.0508 (18)	0.0295 (13)	0.0063 (14)	0.0024 (11)	0.0005 (12)
C6	0.0330 (13)	0.0479 (17)	0.0306 (13)	−0.0024 (12)	0.0024 (11)	−0.0055 (12)
C7	0.0339 (15)	0.069 (3)	0.0476 (19)	−0.0069 (16)	−0.0011 (14)	−0.0065 (18)
C8	0.0444 (19)	0.061 (2)	0.063 (2)	−0.0246 (17)	0.0074 (18)	−0.0163 (19)
C9	0.052 (2)	0.0391 (17)	0.064 (2)	−0.0166 (15)	0.0168 (18)	−0.0104 (16)
C10	0.0373 (14)	0.0345 (14)	0.0361 (14)	−0.0030 (11)	0.0070 (12)	−0.0056 (11)
C11	0.0431 (16)	0.0350 (14)	0.0352 (14)	0.0037 (12)	0.0106 (12)	−0.0022 (11)
C12	0.061 (2)	0.0354 (16)	0.053 (2)	0.0041 (15)	0.0147 (17)	0.0042 (14)
C13	0.068 (3)	0.050 (2)	0.0486 (19)	0.0185 (19)	0.0118 (18)	0.0102 (17)
C14	0.057 (2)	0.062 (3)	0.049 (2)	0.024 (2)	0.0004 (17)	0.0058 (18)
C15	0.0417 (18)	0.055 (2)	0.0487 (19)	0.0085 (15)	0.0002 (15)	−0.0056 (16)
Ni1	0.0447 (2)	0.0389 (2)	0.0450 (2)	0.0022 (2)	0.0074 (2)	−0.0037 (2)
Cl1	0.0557 (5)	0.0396 (4)	0.0668 (6)	−0.0065 (4)	0.0008 (4)	−0.0078 (4)
Cl2	0.0582 (6)	0.0542 (5)	0.0578 (5)	0.0045 (4)	0.0048 (4)	0.0156 (4)
Cl3	0.0789 (7)	0.0736 (7)	0.0531 (5)	0.0027 (6)	0.0231 (5)	−0.0196 (5)
Cl4	0.0395 (4)	0.0623 (7)	0.0851 (7)	0.0057 (4)	0.0075 (4)	−0.0053 (5)
Cl5	0.0413 (4)	0.0435 (5)	0.0823 (7)	−0.0114 (4)	−0.0017 (4)	0.0015 (5)

### Geometric parameters (Å, º)

**Table:**

Ni1—Cl1	2.194 (2)	C7—C8	1.372 (7)
Ni1—Cl2	2.201 (2)	C8—C9	1.392 (6)
Ni1—Cl3	2.188 (2)	C9—C10	1.380 (5)
Ni1—Cl4	2.185 (2)	C10—C11	1.480 (5)
N1—C5	1.347 (5)	C11—C12	1.394 (5)
N1—C1	1.338 (5)	C12—C13	1.390 (7)
N2—C6	1.342 (4)	C13—C14	1.351 (7)
N2—C10	1.338 (4)	C14—C15	1.370 (7)
N3—C15	1.341 (5)	C1—H1	0.9300
N3—C11	1.344 (4)	C2—H2	0.9300
N1—H11	0.8600	C3—H3	0.9300
N2—H22	0.8600	C4—H4	0.9300
N3—H33	0.8600	C7—H7	0.9300
C1—C2	1.387 (7)	C8—H8	0.9300
C2—C3	1.352 (8)	C9—H9	0.9300
C3—C4	1.386 (8)	C12—H12	0.9300
C4—C5	1.384 (6)	C13—H13	0.9300
C5—C6	1.472 (6)	C14—H14	0.9300
C6—C7	1.384 (6)	C15—H15	0.9300
			
Cl1—Ni1—Cl4	109.13 (5)	N2—C10—C9	122.8 (3)
Cl1—Ni1—Cl2	108.08 (5)	N3—C11—C10	116.7 (3)
Cl1—Ni1—Cl3	111.59 (5)	N3—C11—C12	118.2 (3)
Cl3—Ni1—Cl4	108.20 (5)	C10—C11—C12	125.1 (3)
Cl2—Ni1—Cl3	109.57 (5)	C11—C12—C13	118.5 (4)
Cl2—Ni1—Cl4	110.28 (5)	C12—C13—C14	121.3 (4)
C1—N1—C5	124.0 (4)	C13—C14—C15	119.1 (4)
C6—N2—C10	118.9 (3)	N3—C15—C14	119.8 (4)
C11—N3—C15	123.2 (3)	N1—C1—H1	121.00
C5—N1—H11	118.00	C2—C1—H1	121.00
C1—N1—H11	118.00	C1—C2—H2	120.00
C10—N2—H22	121.00	C3—C2—H2	120.00
C6—N2—H22	121.00	C4—C3—H3	120.00
C15—N3—H33	118.00	C2—C3—H3	120.00
C11—N3—H33	118.00	C3—C4—H4	120.00
N1—C1—C2	118.8 (4)	C5—C4—H4	120.00
C1—C2—C3	119.7 (5)	C6—C7—H7	120.00
C2—C3—C4	119.9 (5)	C8—C7—H7	120.00
C3—C4—C5	120.4 (5)	C9—C8—H8	120.00
N1—C5—C4	117.2 (4)	C7—C8—H8	120.00
C4—C5—C6	125.7 (4)	C8—C9—H9	121.00
N1—C5—C6	117.2 (3)	C10—C9—H9	121.00
N2—C6—C5	115.5 (3)	C11—C12—H12	121.00
N2—C6—C7	121.5 (4)	C13—C12—H12	121.00
C5—C6—C7	123.0 (3)	C14—C13—H13	119.00
C6—C7—C8	119.4 (4)	C12—C13—H13	119.00
C7—C8—C9	119.4 (4)	C13—C14—H14	121.00
C8—C9—C10	118.0 (4)	C15—C14—H14	120.00
N2—C10—C11	115.1 (3)	C14—C15—H15	120.00
C9—C10—C11	122.1 (3)	N3—C15—H15	120.00
			
C5—N1—C1—C2	0.4 (6)	C4—C5—C6—N2	175.1 (3)
C1—N1—C5—C4	0.5 (5)	C4—C5—C6—C7	−7.0 (6)
C1—N1—C5—C6	179.7 (3)	N2—C6—C7—C8	0.9 (6)
C10—N2—C6—C5	177.3 (3)	C5—C6—C7—C8	−176.8 (4)
C10—N2—C6—C7	−0.7 (5)	C6—C7—C8—C9	−0.7 (7)
C6—N2—C10—C9	0.2 (5)	C7—C8—C9—C10	0.2 (6)
C6—N2—C10—C11	−179.1 (3)	C8—C9—C10—N2	0.1 (6)
C15—N3—C11—C10	179.5 (3)	C8—C9—C10—C11	179.3 (4)
C15—N3—C11—C12	0.8 (5)	N2—C10—C11—N3	−5.3 (4)
C11—N3—C15—C14	−0.8 (6)	N2—C10—C11—C12	173.4 (3)
N1—C1—C2—C3	−1.0 (7)	C9—C10—C11—N3	175.5 (3)
C1—C2—C3—C4	0.6 (8)	C9—C10—C11—C12	−5.9 (5)
C2—C3—C4—C5	0.4 (7)	N3—C11—C12—C13	0.1 (6)
C3—C4—C5—N1	−1.0 (6)	C10—C11—C12—C13	−178.5 (4)
C3—C4—C5—C6	179.9 (4)	C11—C12—C13—C14	−1.0 (6)
N1—C5—C6—N2	−4.0 (4)	C12—C13—C14—C15	1.0 (7)
N1—C5—C6—C7	173.9 (3)	C13—C14—C15—N3	−0.1 (6)

### Hydrogen-bond geometry (Å, º)

**Table:**

*D*—H···*A*	*D*—H	H···*A*	*D*···*A*	*D*—H···*A*
N1—H11···Cl5	0.86	2.26	3.026 (4)	149
N1—H11···N2	0.86	2.28	2.666 (4)	107
N2—H22···Cl5	0.86	2.67	3.532 (4)	178
N2—H22···N3	0.86	2.29	2.654 (4)	106
N3—H33···Cl5	0.86	2.25	3.010 (4)	148
C14—H14···Cl5^i^	0.93	2.78	3.421 (6)	127

Symmetry code: (i) −*x*, *y*+1/2, −*z*.
